# Longitudinal Changes of Tongue Thickness and Tongue Pressure in Neuromuscular Disorders

**DOI:** 10.1186/s12883-021-02225-5

**Published:** 2021-08-05

**Authors:** George Umemoto, Shinsuke Fujioka, Hajime Arahata, Nobutaka Sakae, Naokazu Sasagasako, Mine Toda, Hirokazu Furuya, Yoshio Tsuboi

**Affiliations:** 1 Swallowing Disorders Center, Fukuoka University Hospital, 7-45-1 Nanakuma, Jonan-ku, Fukuoka, 814-0180 Japan; 2 Department of Neurology, Neuro-Muscular Center, NHO Omuta National Hospital, Fukuoka, Japan; 3 Department of Neurology, Faculty of Medicine, Fukuoka University, Fukuoka, Japan; 4 Department of Clinical Nutrition & Food services, NHO Omuta National Hospital, Fukuoka, Japan; 5 Department of Neurology, Faculty of Medicine, Kochi University, Kochi, Japan

**Keywords:** Tongue thickness, Tongue pressure, Amyotrophic lateral sclerosis, Myotonic dystrophy type 1, Duchenne muscular dystrophy

## Abstract

**Background:**

Swallowing dysfunction is related to major cause of adverse events and an indicator of shorter survival among patients with neuromuscular disorders (NMD). It is critical to assess the swallowing function during disease progression, however, there are limited tools that can easily evaluate swallowing function without using videofluoroscopic or videoendoscopic examination. Here, we evaluated the longitudinal changes in tongue thickness (TT) and maximum tongue pressure (MTP) among patients with amyotrophic lateral sclerosis (ALS), myotonic dystrophy type 1 (DM1), and Duchenne muscular dystrophy (DMD).

**Methods:**

Between 2010 and 2020, TT and MTP were measured from 21 ALS, 30 DM1, and 14 DMD patients (mean ages of 66.9, 44.5, and 21.4 years, respectively) at intervals of more than half a year. TT was measured, by ultrasonography, as the distance from the mylohyoid muscle raphe to the tongue dorsum, and MTP was determined by measuring the maximum compression on a small balloon when pressing the tongue against the palate. Then we examined the relationship between these evaluations and patient background and swallowing function.

**Results:**

Mean follow-up periods were 24.0 months in the ALS group, 47.2 months in the DM1group, and 61.1 months in the DMD group. The DMD group demonstrated larger first TT than the other groups, while the DM1 group had lower first MTP than the ALS group. The ALS group showed a greater average monthly reduction in mean TT than the DM1 group and greater monthly reductions in mean body weight (BW) and MTP than the other groups. Significant differences between the first and last BW, TT, and MTP measures were found only in the ALS group.

**Conclusions:**

This study suggests that ALS is associated with more rapid degeneration of tongue function over several years compared to DMD and DM1.

## Background

Patients with neuromuscular disorders (NMD) often experience problems with swallowing during the course of the illness. They require a periodic video fluoroscopic swallowing study (VFSS) or fiberoptic endoscopic evaluation of swallowing (FEES) to reduce the risk of aspiration pneumonia and assess the need for appropriate nutritional support. However, these tests require equipment and can only be evaluated in specialized facilities, and it is difficult to assess tongue function, using VFSS or FEES, quantitatively. Clinically, tongue function is determined by multiple characteristics, including mobility, shape, and posture. In the oral phase, tongue dysfunction may induce inadequate “bolus formation”, “mastication”, and “premature bolus loss” which cause pharyngeal residue or penetration/ aspiration. In NMD, dysphagia may result from tongue muscle weakness as well morphological changes due to muscle hypertrophy or atrophy. For instance, an enlarged tongue is frequently observed in Duchenne muscular dystrophy (DMD) while tongue atrophy is common in amyotrophic lateral sclerosis (ALS). Tongue hypertrophy which causes chewing and swallowing difficulties needs adequate nutritional support in DMD [1, 2], and tongue muscle deficit is the major factor causing dysphagia in ALS [3]. Therefore, monitoring these changes is critical for clinical management during disease progression.

In our previous study, we evaluated the relationship between tongue thickness (TT) and strength in NMD patients at a single time point [4] and found that TT was significantly greater in DMD patients, while maximum tongue pressure (MTP) was lower in myotonic dystrophy type 1 (DM1) patients compared to other NMD groups. Moreover, that study revealed a significant correlation between TT and MTP in ALS. These findings indicate that association between TT, MTP, and dysphagia varies depending on the type of NMD. However, longitudinal changes of TT and MTP of NMD patients except ALS have yet to be studied [5]. TT and MTP were reported to be associated with oral preparatory and swallowing efficacy [6, 7] and this information is critical for evaluating aspiration and malnutrition risk over time. We hypothesized that the progression rate of tongue dysfunction represented by TT and MTP could be different depending on NMD and influence on nutrition condition, weight loss. This study aimed to characterize the longitudinal changes in TT and MTP as well as body weight (BW) and find a clue to establish disorder-specific treatment plans for dysphagia in ALS, DM1, and DMD patients.

## Methods

### Subjects

Between 2010 January to and 2020 December, TT and MTP were measured from 21 ALS patients (9 males and 12 females; mean age, 66.9 years), 30 DM1 patients (16 males and 14 females; mean age, 44.5 years), and 14 male DMD patients (mean age, 21.4 years) at the Department of Neurology, Neuro-Muscular Center, NHO Omuta National Hospital, Omuta City, Japan. Patients received multiple TT and TP measurements at intervals of more than half a year. All DMD and DM1 cases were confirmed by genetic analysis after clinical diagnosis, and all ALS patients were clinically diagnosed as probable or definite by neurologists specializing in ALS, based on Awaji criteria [8]. Furthermore, all ALS patients were monitored clinically for more than one year before enrolling in this study. These 21 patients were divided into 14 bulbar onset and 7 limb onset subgroups for analysis. TT and MTP measurements and VFSS are conducted as part of standard examinations in the NHO Omuta National Hospital. The ethics committee (or institutional review board) of the NHO Omuta National Hospital approved the informed consent procedure to obtain through an opt-out provision on the study website and the analysis of the examinations in this study. Physical examinations were conducted on all patients, including measures of BW, height, and body mass index (BMI).

### Functional Oral intake scale

The multidisciplinary team classified diet type into seven levels based on the functional oral intake scale (FOIS) [9]: Level 0, tube dependent; Level 1, no oral intake; Level 2, tube dependent with minimal/inconsistent oral intake; Level 3, tube supplementation but consistent oral intake; Level 4, total oral intake of a single consistency; Level 5, total oral intake of multiple consistencies requiring special preparation; Level 6, total oral intake with no special preparation, but with specific foods or liquid items avoided; and Level 7, total oral intake with no restrictions.

### Tongue thickness

**Fig. 1:**
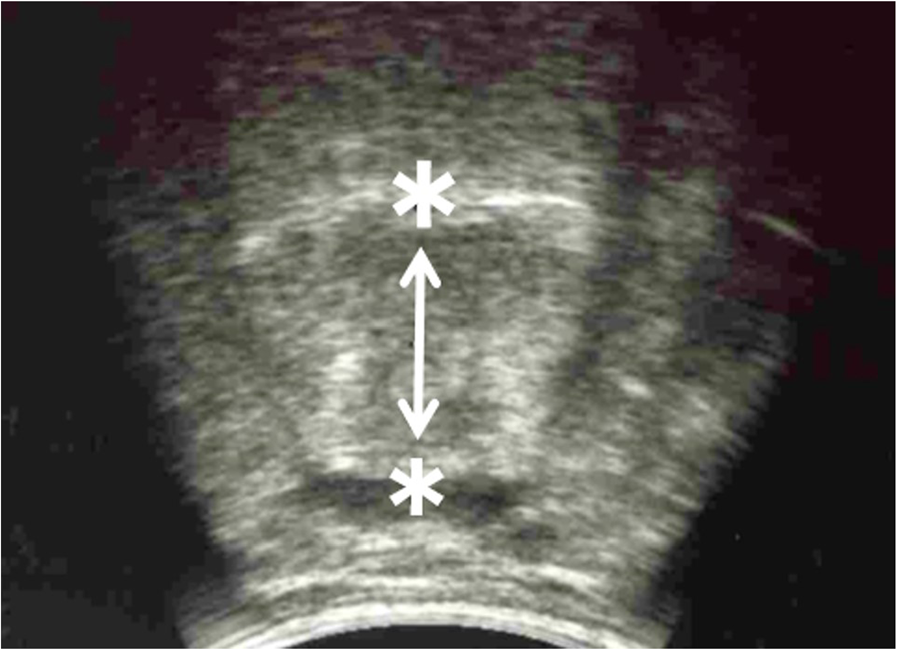
Tongue thickness measured by ultrasonography. Tongue thickness was defined as the distance from the upper boundary of the mylohyoid raphe to the upper surface of the tongue, including the geniophyoid and genioglossus muscles

TT was measured from the mylohyoid muscle raphe to the upper surface of the tongue (Fig. 1) using a Fukuda Denshi UF-550XTD ultrasound system (Fukuda Denshi, Tokyo, Japan). The transducer and equipment settings were monitored constantly to obtain optimal ultrasound images for quantitative analysis [10]. Measurements were performed three times for the submental muscle group, and the average value was calculated. The TT was measured in a sitting position with relaxed normal head and neck posture and relaxed closed mouth. Excessive pressure on the skin during scanning was avoided by the generous use of contact gel.

### Measurement of tongue strength

Tongue strength was measured as MTP using a handy probe consisting of a small balloon pressurized with air to 19.6 kPa (JM-TPM; JMS, Hiroshima, Japan) [11]. Each participant was required to compress the balloon against the palate using the tongue for approximately 7 s while applying maximum effort. The resulting increase in balloon inner pressure was measured and recorded as MTP. This test was repeated three times, and the mean value was obtained for analysis.

### Data analysis

Mean BW, TT, and MTP were compared among groups using Steel-Dwass test. Pearson correlation coefficients were calculated to assess the relationships among BW, TT, and MTP. The first and last BW, MTP, and TT measurements were compared by the Wilcoxon signed-rank sum tests. All statistical analyses were performed using SPSS 13.0 J for Windows (SPSS, Inc., Chicago, IL, USA). *p* < 0.05 (two tailed) was considered statistically significant for all tests.

## Results

Patients clinical characteristics are summarized in Table 1. As expected in the ALS group, mean age was higher, while follow-up duration was significantly shorter than the other groups. Mean FOIS at the first measurement decreased by the last one, from 6.3 ± 1.3 to 4.6 ± 1.5 in the ALS group, from 6.1 ± 1.2 to 4.9 ± 1.3 in the DM1 group, from 5.3 ± 1.3 to 3.9 ± 1.8 in the DMD group. At the first measurement, more than 80% of ALS and DM1 patients ate with a few restrictions but 43% of DMD patients did dysphagia diets. The first mean BW was significantly lower in the DMD group than the ALS and the DM1 groups (*p* < 0.05, *p* < 0.01, respectively). The mean first TT was larger in the DMD group than the other groups (*p* < 0.01), while MTP was lower in the DM1 group than the ALS group (*p* < 0.05).

**Table 1:**
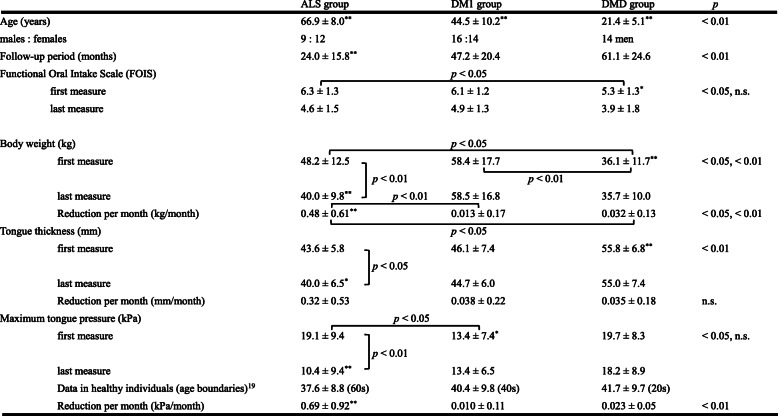
Comparison of the three groups’ patient characteristics, body weight, maximum tongue pressure and tongue thickness

^*^p < 0.05, ^**^p < 0.01. Differences in age, FOIS, body weight, tongue pressure, and tongue thickness were analyzed using Steel-Dwass test. Differences between the first and last measures of body weight, tongue pressure and tongue thickness were analyzed using the Wilcoxon signed-rank sum tests

**Fig. 2:**
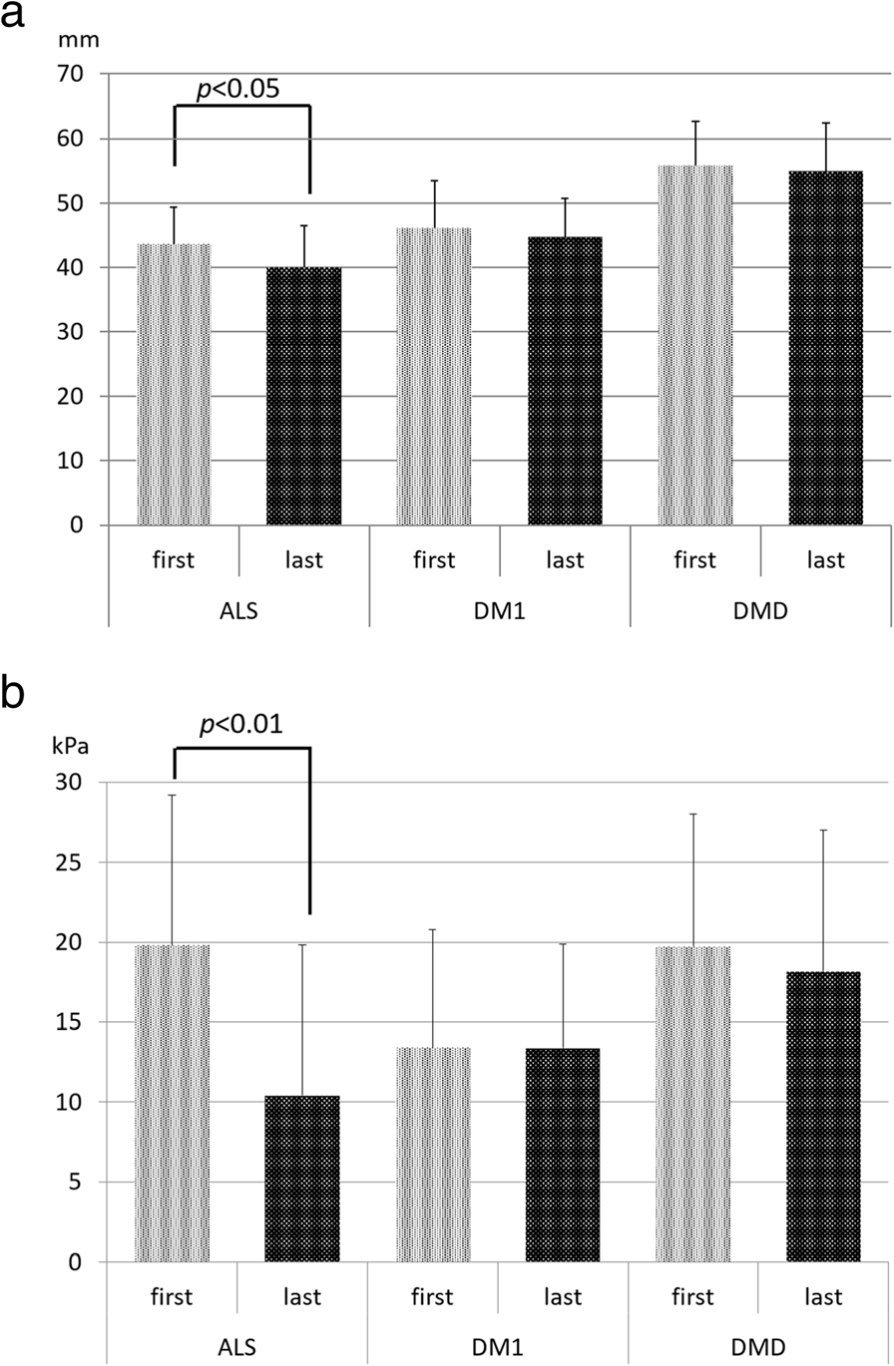
**a** Changes in tongue thickness (TT) among the three disease groups. The ALS group showed significant differences in TT between the first and last measurements (*p* < 0.05). **b** Changes in maximum tongue pressure (MTP) among the three disease groups. The ALS group showed significant differences in MTP between the first and last measurements (*p* < 0.01)

However, the ALS group demonstrated the greater average monthly reduction in mean BW than the DM1 and DMD groups (*p* < 0.01, *p* < 0.05, respectively), and the greater average monthly reduction in mean MTP compared to both the DM1 and DMD groups (*p* < 0.01). The ALS group marked the greater average monthly reduction in mean TT but no significant difference from the other two groups. Moreover, only the ALS group showed significant differences in BW, TT and MTP between the first and last measurements (*p* < 0.01, *p* < 0.05, *p* < 0.01, respectively) (Fig. 2a, b).

**Fig. 3:**
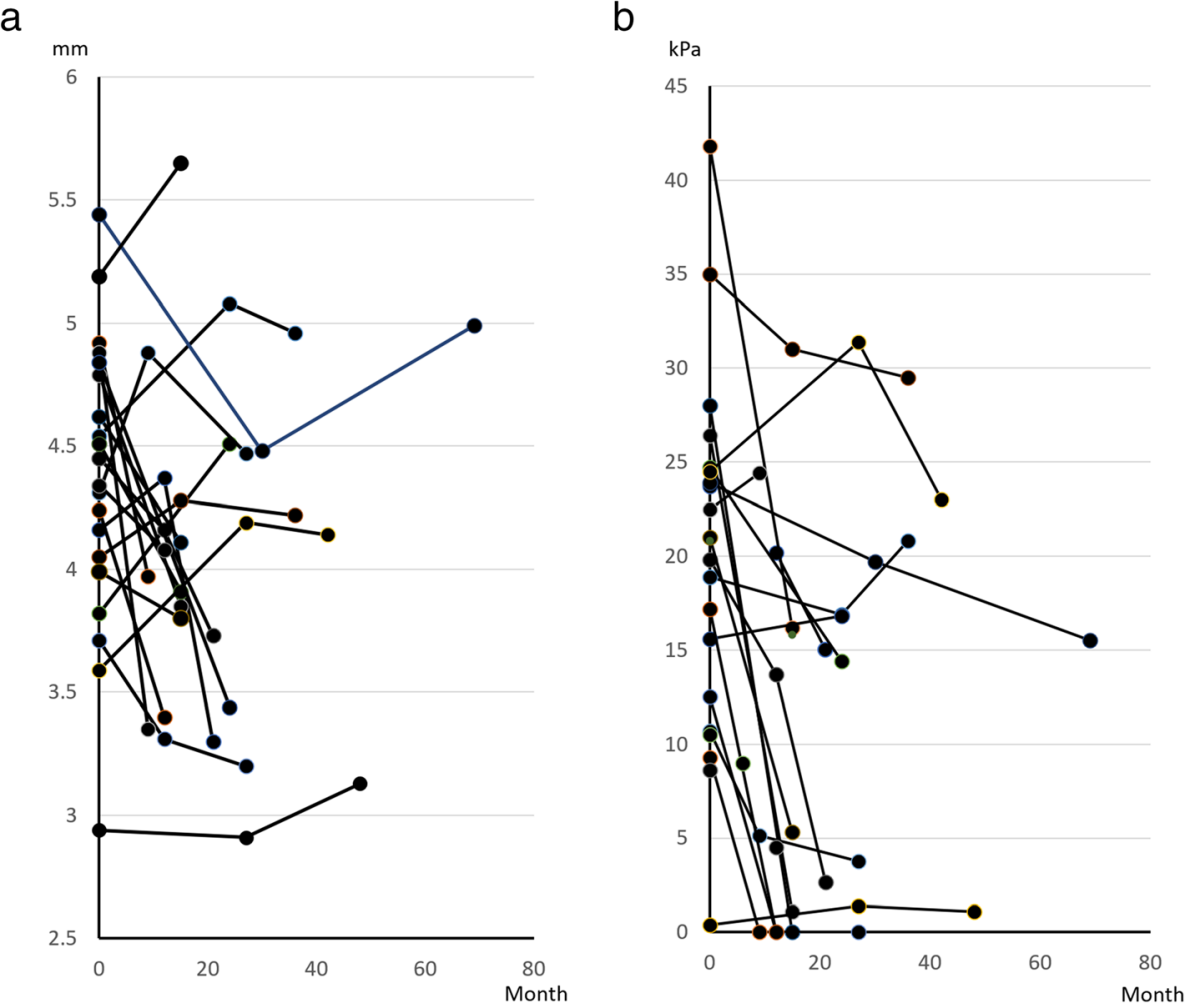
**a** Change in tongue thickness (TT) from the first to the last measurement in the ALS group. **b** Change in maximum tongue pressure (MTP) from the first to the last measurement in the ALS group

Analysis of individual subjects revealed TT and MTP were reduced in 15 of 21 ALS patients (Fig. 3a, b). Subgroup analysis revealed no significant difference in the incidence of reduced TT between bulbar and limb onset patients, while none of the ALS patients with bulbar onset had a substantial reduction in MTP. There were no significant correlations among reduction rates of BW, MTP, and TT in any NMD group.

## Discussion

In our previous cross-sectional study, we evaluated the relationship between TT and MTP as a strength metric in DMD, DM1, and ALS patients. There was higher TT in the DMD group, lower MTP in the DM1 group, and a significant correlation between TT and MTP in the ALS group [4]. Thirteen of 21 ALS patients, 19 of 30 DM1 patients, 11 of 14 male DMD patients in this study overlapped with the previous one, and measurement values at the first measure of this study were close to the previous ones. In another study, we also revealed the distinctive features of dysphagia in DM1 and DMD patients using VFSS, hyoid bone movement during the pharyngeal phase of swallowing (excursion), or pharyngeal residue measurement [12], which also demonstrated distinct abnormalities in swallowing muscle function between DM1 and DMD. The study also showed the correlation between TT and BW, but not MTP, in the DM1 group [4], suggesting that, in DM1 patients, TT was associated with malnutrition and concomitant weight loss. However, long-term tongue muscle weakness is also thought to promote malnutrition, and further loss of muscle mass may lead to additional TT and BW reductions [13]. Therefore, we assessed TT, MTP, and BW at multiple times during disease progression in this study.

Van Den Engel-Hoek et al. reported that TT can be assessed, conveniently and reproducibly, in DMD patients using ultrasound [10, 14]. More recently, they reported increased tongue hypertrophy and dystrophic changes in masticatory muscles but did not report changes in tongue muscles [1]. Although we found “tongue pseudohypertrophy” in DMD patients by ultrasound assessment, there were no significant longitudinal changes as in the ALS group. We suggest that, in terms of TT progression, after the early stage of DMD that tongue muscle tissue is replaced by connective tissue or fat [15], the progression rate will become slower.

On the other hand, progressive bulbar palsy is rapidly linked to dysphagia, malnutrition, and BW loss which leads to poor survival time in ALS, but BW loss could be caused by rising energy consumption due to impairment of respiratory function [16]. Poor tongue strength is independently associated with shorter survival time in ALS patients [17], so measuring TT and MTP is critical for prognosis and adjustment of treatment. Tamburrini et al. examined tongue movement function among ALS patients using both ultrasonography and VFSS, but they did not conduct objective tongue atrophy assessment [18]. Nakamori et al. suggested an association between TT, disease progression, and tongue dysfunction [6], consistent with our previous study showing a significant correlation between TT and MTP in ALS [4]. The mean TT value of ALS patients in their study (41.9 ± 4.0 mm) was similar to our last measurement (40.0 ± 6.5 mm), while the mean TT value of their control volunteers (44.8 ± 3.0 mm) was closer to the first value in our ALS group (42.8 ± 6.8 mm), strongly suggesting tongue atrophy progressed during the roughly 2-year follow-up period. Nakamori et al. also found reduced TT over 15 months. In accordance with their study, the ALS group also included patients without TT reduction (Fig. 3a), possibly due to differences in time from onset or rate of disease progression between bulbar or limb onset types. The patients with limb onset types showed a greater reduction MTP than bulbar onset patients and limb onset patients were likely in an advanced clinical stage. Increased TT in few patients may be caused by measurement error which is expected about 10% one way or the other, due to the position of the ultrasound imaging probe. Alternatively, we found no association between TT and BW, in contrast to the linear association between TT and BMI reported by Nakamori et al. In our previous study, bulbar onset patients showed a stronger correlation between TT and MTP than limb onset patients, suggesting these measures especially important for monitoring bulbar paralysis patients [4], especially during the early clinical stages.

This study has several limitations, most notably the differences in mean age among groups, which is known to influence MTP [19]. However, age matching of DMD, ALS, and DM1 is difficult due to the differences in age at onset and course. Moreover, differences in gender or evaluation periods are also difficult to unify in the three groups. There are only male DMD patients and we considered that gender differences did not have a big effect on temporal changes of TT and MTP. The periodic evaluations were ordered depending on the disease progression and ALS patients underwent more examinations in the same term. In the initial stage, we often examined them once a year but increased the frequency of examinations depending on the deterioration of symptoms. Unfortunately, there is no uniform standard to assess the functional level or disease stage for NMD patients and ADL scales are not correlate well with their eating ability. Instead of functional level or disease stage, we used the score of FOIS to show their eating abilities. In the future, a further investigation with enough sample size is requested in order to confirm the distinctive characteristics of tongue dysfunction progression in NMD and to establish disorder-specific treatment plans for dysphagia. Nonetheless, these results suggest distinct dysphagia pathomechanisms and progression features of among neurogenic and myogenic disorder patients.

This longitudinal study revealed more rapid loss of tongue thickness and strength in ALS patients than DMD and DM1. Only the ALS patients also exhibited substantial weight loss over the observation period, a change strongly associated with shorter survival [16]. On the other hand, DM1 and DMD patients may show only slowly developing tongue dysfunction in the short term, although DM1 may have lower tongue strength and DMD patients enlarged tongues, respectively, both of which can contribute to dysphagia. These findings suggest that regular re-evaluation of TT and MTP could provide valuable information on tongue dysfunction progression in NMD.

## Conclusions

ALS is associated with a more rapid progression of tongue dysfunction over several years than DMD and DM1. We can conveniently identify the progression of tongue dysfunction based on temporal changes of TT and MTP. TT and MTP are more helpful to examine the relationships between the progress of tongue dysfunction and NMD than VFSS and FESS.

## Data Availability

The datasets used and/or analyzed during the current study available from the corresponding author on reasonable request.

## Abbreviations

ALS: Amyotrophic lateral sclerosis
BW: Body weight
BMI: Body mass index
DMD: Duchenne muscular dystrophy
DM1: Myotonic dystrophy type 1
FEES: Fiberoptic endoscopic evaluation of swallowing
FOIS: Functional oral intake scale
MTP: Maximum tongue pressure
NMD: Neuromuscular disorders
TT: Tongue thickness
VFSS: video fluoroscopic swallowing study
